# Association of group-level segregation with cardiovascular health in older adults: an analysis of data from the Korean Social Life, Health, and Aging Project

**DOI:** 10.4178/epih.e2023041

**Published:** 2023-04-04

**Authors:** Sung-Ha Lee, Hyeok-Hee Lee, Kiho Sung, Yoosik Youm, Hyeon Chang Kim

**Affiliations:** 1 Center for Happiness Studies, Seoul National University, Seoul, Korea; 2 Department of Preventive Medicine, Yonsei University College of Medicine, Seoul, Korea; 3 Department of Internal Medicine, Yonsei University College of Medicine, Seoul, Korea; 4 Department of Sociology, Yonsei University, Seoul, Korea

**Keywords:** Social segregation, Cardiovascular disease, Heart disease risk factors, Social network analysis, Cohort studies

## Abstract

**OBJECTIVES:**

The adverse health effects of individual-level social isolation (e.g., perceived loneliness) have been well documented in older adults. However, little is known about the impact of collective-level social isolation on health outcomes. We sought to examine the association of group-level segregation with cardiovascular health (CVH) in older adults.

**METHODS:**

From the prospective Korean Social Life, Health, and Aging Project database, we identified 528 community-dwelling older adults who were aged ≥60 years or were married to those aged ≥60 years. Participants who belonged to smaller social groups separate from the major social group were defined as group-level-segregated. The CVH score was calculated as the number of ideal non-dietary CVH metrics (0-6), as modified from the American Heart Association’s Life’s Simple 7. Using ordinal logistic regression models, we assessed cross-sectional and longitudinal associations between group-level segregation and CVH.

**RESULTS:**

Of the 528 participants (mean age, 71.7 years; 60.0% female), 108 (20.5%) were segregated at baseline. In the cross-sectional analysis, group-level segregation was significantly associated with lower odds of having a higher CVH score at baseline after adjusting for socio-demographic factors and cognitive function (odds ratio [OR], 0.64; 95% confidence interval [CI], 0.43 to 0.95). Among 274 participants who completed an 8-year follow-up, group-level segregation at baseline was marginally associated with lower odds of having a higher CVH score at 8 years (OR, 0.49; 95% CI, 0.24 to 1.02).

**CONCLUSIONS:**

Group-level segregation was associated with worse CVH. These findings imply that the social network structure of a community may influence its members’ health status.

## GRAPHICAL ABSTRACT

[Fig f4-epih-45-e2023041]

## INTRODUCTION

As social beings, humans must maintain social relationships for survival and well-being. A growing body of evidence indicates that insufficient social connections lead to higher mortality, suggesting that social relationships are crucial for health and longevity [1-3]. In particular, social isolation has repeatedly been reported as a risk factor for premature mortality [3-5]. Among the possible mechanisms linking social disconnection to morbidity and mortality, cardiovascular health (CVH) has emerged as a significant mediator through which social relationships might impact health outcomes [6-9]. Previous studies have shown that individuals with a higher level of loneliness or social isolation exhibit greater risks of hypertension, stroke, coronary heart disease, and myocardial infarction [7,8,10]. Given the multifactorial nature of social relationships, holistic and integrative approaches may be required to investigate their impact on health outcomes.

A substantial number of studies have focused primarily on individual-level social isolation measures (e.g., lack of social contact or perceived loneliness) concerning health outcomes. In contrast, only a few studies have investigated the prognostic implications of group-level segregation [11-14]. In a recent study examining group-level segregation among individuals of the same race within a single village, segregated older adults exhibited significantly worse self-rated health and higher all-cause mortality after 8 years of follow-up [13,14]. Although these studies have raised awareness of the potential health effects of community-level social relationship factors, the mechanisms underlying the association between group-level segregation and health outcomes remain elusive.

Using the prospective Korean Social Life, Health, and Aging Project (KSHAP) database, which includes information on the complete social network structure of a single village in Korea, we examined (1) the cross-sectional association between group-level segregation and CVH at baseline; (2) temporal changes in group-level segregation status and CVH; and (3) the longitudinal association between group-level segregation and CVH at follow-up.

## MATERIALS AND METHODS

### Data source

The KSHAP is a prospective cohort study designed to examine social activity, health-related factors, and aging in Korean older adults. All inhabitants of Township K in Ganghwa Island, Korea who were aged ≥ 60 years or were married to those aged ≥ 60 years were recruited.

Township K is one of the 12 townships of Ganghwa Island, the fourth largest island in Korea. The township is a rural community located in the northernmost part of the island, which has been connected to the mainland by bridges since 1970. Most of the inhabitants engage in farming, and nearly half are aged over 60 [15]. The cohort has collected information on participants’ social network structure biennially since its first wave in 2011 and conducted health examinations in 2011 (baseline) and 2019 (follow-up).

### Study population

A total of 860 participants were recruited in the KSHAP at baseline (2011). Of the 860 individuals in the target population, 814 (94.7%) completed an enumeration survey in the first wave of the KSHAP between December 2011 and July 2012. Among them, 698 participants underwent additional health examinations and were included in the KSHAP-health examination cohort [16]. After excluding 169 participants and 1 participant without available data on the Timed Up-and-Go (TUG) test and educational attainment, respectively, the final study cohort comprised 528 participants. For the longitudinal analysis, the analytic sample was further limited to 274 participants who had complete data for the aforementioned variables at both baseline (2011) and follow-up (2019).

### Group-level segregation

**Figure 1.**
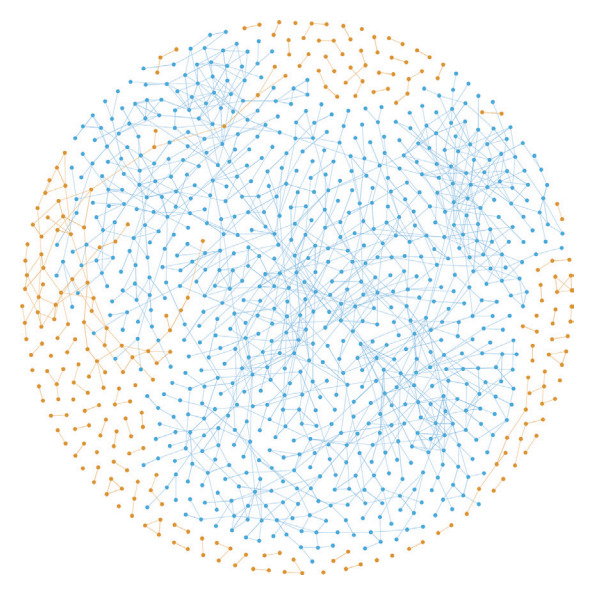
Complete social network map of Township K at baseline. Orange and blue dots denote segregated and integrated participants, respectively.

Group-level segregation status was assessed using a complete social network analysis of Township K [13,17]. In summary, social network nodes were constructed using the information from participants naming up to 6 discussion partners who resided in the same town. Next, the “component” referring to a maximally connected subnetwork was constructed as previously described [13,14]. Any pair of individuals could be reachable either directly or indirectly within the same component, whereas individuals belonging to different components were separated and disconnected. Figure 1 depicts the components constructed in Township K; the most significant component to which the majority of the residents belonged included 672 nodes (blue dots). Group-level segregation was defined as belonging to smaller components separate from the major component (orange dots; Figure 1).

### Cardiovascular health score and covariables

Each participant’s CVH score was calculated as the number of ideal non-dietary CVH metrics (0-6), as modified from the American Heart Association’s Life’s Simple 7 [18]. The 6 CVH metrics included in the score were smoking, body mass index (BMI), blood pressure (BP), fasting glucose, total cholesterol, and physical function. A higher CVH score reflects better CVH status. Smoking status was assessed as never, former, or current smoking; never or former smoking was defined as the ideal smoking status. BMI was calculated by dividing the weight in kilograms by height in meters squared, and a BMI between 18.5 kg/m^2^ and 22.9 kg/m^2^ was defined as ideal BMI status. BP was measured using an oscillometric sphygmomanometer after 5 minutes of rest in the sitting position. Three BP measurements were obtained at 1-minute intervals, and the average of the 3 measurements was used as each participant’s BP; an untreated BP < 120/80 mmHg was defined as ideal BP status. Fasting glucose and total cholesterol levels were measured from blood samples collected after an 8-hour fasting period; an untreated fasting glucose level < 100 mg/dL and a total cholesterol level < 200 mg/dL were defined as ideal fasting glucose and total cholesterol status, respectively. We assessed participants’ physical function using the TUG test, as we lacked detailed information on their physical activity levels. During the test, a medical staff measured the time it took for the participants to rise from an armchair, walk a distance of 3 m, turn, walk back to the chair, and sit down [19]; a TUG time < 13.5 seconds was defined as ideal physical function status. The TUG time has been reported as a good predictor of functional mobility and frailty in older adults [20].

Data on participants’ demographics, educational attainment (elementary school or less, middle school, high school, and college or higher), social network size, marital status (living with a spouse or not), Mini-Mental State Examination for Dementia Screening (MMSE) score, and household income were also collected.

### Statistical analysis

Baseline characteristics were presented as mean± standard deviation or number (%) and compared using the independent t-test or chi-square test. In the cross-sectional analysis, we examined the association of group-level segregation with the CVH score at baseline using ordinal logistic regression models, treating the CVH score as an ordinal variable from 0 to 6. The “Polr” function of the MASS package in R was used for the analyses. Potential confounders were adjusted stepwise: model 1 included age, sex, and educational attainment; model 2 was further adjusted for social network size and marital status; and model 3 was further adjusted for the MMSE score.

In the longitudinal analysis, we assessed the temporal changes in group-level segregation status and CVH score among 274 participants who completed an 8-year follow-up. We also investigated the association of group-level segregation at baseline with CVH score at 8 years using the same regression models mentioned above, all of which were further adjusted for the baseline CVH score.

As sensitivity analyses, we repeated our main regression analyses after further adjusting for household income or depressive symptoms assessed by the Center for Epidemiologic Studies Depression scale in the subset of participants who had available information. All statistical analyses were conducted using R version 4.0.3 (R Foundation for Statistical Computing, Vienna, Austria).

### Ethics statement

This study complied with the Declaration of Helsinki, and the study protocol was approved by the Institutional Review Board of Yonsei University, Seoul, Korea (YUIRB-2011-012-01; 7001988-202111-HR-505-04). Written informed consent was obtained from all participants.

## RESULTS

### Baseline characteristics of the participants

Of the 528 participants (mean age, 71.7 years; 60.0% female), 108 (20.5%) displayed social segregation at baseline. The segregated individuals were younger, were more highly educated, had smaller social networks, and lived with their spouses less frequently than those who were integrated (Table 1).

### Association of group-level segregation with the cardiovascular health score at baseline

Group-level segregation was significantly associated with reduced odds of having a higher CVH score at baseline after adjusting for demographics and educational attainment (model 1: odds ratio [OR], 0.65; 95% confidence interval [CI], 0.44 to 0.95). The association remained significant after further adjusting for social network size and marital status (model 2: OR, 0.61; 95% CI, 0.41 to 0.90), and MMSE score (model 3: OR, 0.64; 95% CI, 0.43 to 0.95) (Table 2). The findings were generally consistent when we additionally adjusted for household income (n=431; OR, 0.69; 95% CI, 0.43 to 1.11) or depressive symptoms (n=521; OR, 0.64; 95% CI, 0.44 to 0.96) in the sensitivity analyses.

### Temporal changes in group-level segregation status and cardiovascular health score

Of the 274 participants (mean age, 69.7 years; 61.7% female) who completed an 8-year follow-up, 34 (12.4%) showed social segregation at baseline (Supplementary Material 1). Similar to the participants in cross-sectional analysis, the segregated individuals were younger and more highly educated than integrated individuals. However, the 2 groups did not differ regarding social network size, marital status, or cognitive function (Supplementary Material 1).

**Figure 2.**
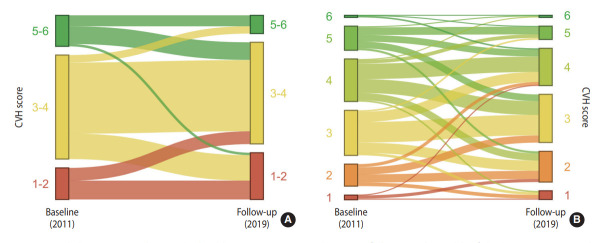
Temporal changes in cardiovascular health (CVH) scores over the 8-year follow-up. The width of the line is proportional to the number of participants. The CVH scores were classified into three groups: 1-2, 3-4, and 5-6 (A) and six groups (B).

**Figure 3.**
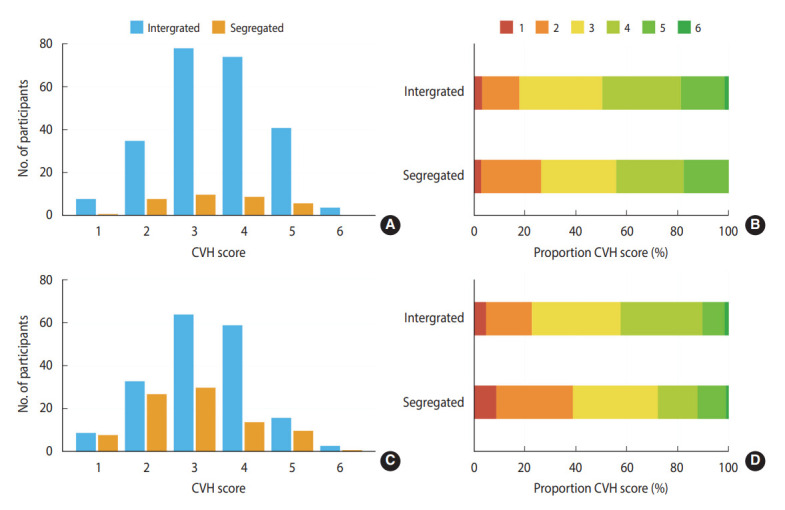
Cardiovascular health (CVH) score distributions. The distribution of CVH scores at baseline (A, B) and at the 8-year follow-up (C, D).

Over the follow-up, the proportion of segregated individuals increased from 12.4% to 32.8% (p<0.001; Table 3), and the CVH score decreased, remained the same, and increased in 40.1%, 37.6%, and 22.3% of the participants, respectively (Figure 2 and Table 3). When each metric was analyzed separately, the proportion of participants with ideal fasting glucose decreased from 76.3% to 66.4% (p=0.011), and that of participants with ideal physical function decreased from 74.8% to 58.0% (p<0.001; Table 3). The distribution of CVH scores at baseline and at 8 years among those who completed the follow-up is described in Figure 3.

### Association of group-level segregation at baseline with cardiovascular health score at follow-up

Group-level segregation at baseline was marginally associated with reduced odds of having a higher CVH score at 8 years after adjusting for baseline CVH score, demographics, educational attainment, social network size, marital status, and MMSE score (model 3: OR, 0.49, 95% CI, 0.24 to 1.02) (Table 4). The findings were broadly similar when we additionally adjusted for household income (n=231; OR, 0.44; 95% CI, 0.18 to 1.06) or depressive symptoms (n=270; OR, 0.50, 95% CI, 0.23 to 1.02) in the sensitivity analyses.

## DISCUSSION

This prospective cohort study showed that community-level social isolation was significantly associated with worse CVH among Korean older adults. Segregated participants within small groups whose network did not reach the whole village exhibited worse CVH profiles than those who were integrated. Moreover, this negative association between group-level segregation and CVH persisted after 8 years, implying that socially isolated individuals, in general, remained unhealthy unless they were specially cared for or received an intervention of some sort. The associations of group-level segregation with individual CVH metrics varied to some extent, especially for smoking and total cholesterol (Supplemental Material 2). However, the analyses were largely underpowered to draw any definitive conclusions.

Our findings are in accordance with previous studies demonstrating the associations of neighborhood-level segregation with cardiovascular diseases and mortality [21-23]. However, those studies primarily focused on African Americans and investigated how racial or ethnic residential segregation contributed to health disparities. Non-social differences between races or ethnicities (e.g., genetic predisposition and racial discrimination) may have contributed to the relationship between segregation and health outcomes. Further evidence on the direct connection between social aspects of segregation and an individual’s health status has therefore been warranted.

The present study focused on whether group-level segregation in a homogeneous ethnic group can impact health outcomes. Utilizing a single racial group and complete social network structure of an entire town, we showed that group-level segregation was associated with worse CVH at both baseline and 8 years. Herein, we speculate that 2 possible pathways might link group-level segregation and CVH. First, segregated older adults may be less likely to have access to communication resources disseminated through local community networks, which may restrict their ability to obtain sufficient health information for maintaining ideal CVH. Among racially segregated African Americans and Latinos in the United States, limited access to community-based communication infrastructure was associated with a reduced chance of obtaining knowledge about the detection and prevention of chronic diseases [24]. In this light, encouraging segregated older adults to engage in community-level programs, including local walking groups and community organization-based exercise, might be an effective intervention for promoting CVH [25,26]. This appears to be a viable approach, given that nearly 70% of Korean older adults in rural areas regularly attend senior community centers that offer social and recreational activities as well as health and wellness programs [27]. The finding that segregated older adults were younger and more educated than their integrated counterparts also renders such an approach more feasible. Second, social segregation can increase the level of psychological distress, which is a significant risk factor for poor CVH. Previous studies have suggested that psychological stress may, at least partly, mediate the association between the level of segregation and blood pressure [23,28]. It can be argued that segregated individuals may lack a sense of belonging to the community or lack community-level social support, which can elevate stress levels and subsequently lead to an increased risk of cardiovascular disease [29,30].

A growing body of evidence has linked health outcomes with individual-level social disconnection, such as loneliness, which is a subjective experience of social isolation [10,31-33]. However, less attention has been paid to the impact of group-level or society-level isolation on individuals’ health outcomes. The present study extends the existing literature by exploring the potential impact of community-level social relationship factors obtained from social network analysis on CVH profiles. Taken together with the recent finding that group-level segregation is a strong predictor of mortality [13], our results imply that CVH may be a potential mediator between segregation and mortality. However, a mechanistic understanding of the observed findings will require further comprehensive investigations, which should include social, structural, and functional factors that determine social connection or health status [31,34].

Our findings should be interpreted in light of several limitations. First, a causal relationship between group-level segregation and worse CVH could not be established due to the observational nature of the study. Second, despite adjusting for major covariables, including demographic characteristics, social factors, and cognitive function, the possibility of residual confounding still exists. In particular, unmeasured confounders, such as nutritional intake, physical activity, and genetic predisposition, may have affected the association between group-level segregation and CVH. Third, given the cohort’s low retention rate (52%) over the follow-up, we cannot exclude the possibility of selection bias in our longitudinal analysis. As a matter of fact, the participants who completed the follow-up were significantly younger and, interestingly, less segregated than those who were lost to follow-up (Supplemental Material 3). It is possible that group-level segregation was a factor that influenced the retention rate. Fourth, although group-level segregation was consistently associated with worse CVH in various analyses, its associations with individual CVH metrics varied to some extent. Further research is needed to firmly establish the impact of group-level segregation on each CVH metric. Fifth, each component of the CVH score may have a differential influence on cardiovascular disease risk. However, as the main purpose of the CVH score is to monitor how well an individual or a population manages their cardiovascular risk profile rather than predicting the absolute risk of developing cardiovascular disease, we focused on the number of ideal CVH metrics without considering the differential impact of each component [18]. Finally, the study cohort was composed entirely of Korean older adults; whether the findings can be generalized to other ethnic or racial populations or age groups should be determined in future studies.

In older adults, group-level segregation was associated with worse CVH at both baseline and 8 years. These findings underscore the importance of community-level social isolation as one of the potential determinants of an individual’s health status that should be considered when developing future health promotion interventions for older adults. Further studies are warranted to demonstrate the potential mechanisms by which group-level segregation affects an individual’s health outcomes.

## Figures and Tables

**Figure f4-epih-45-e2023041:**
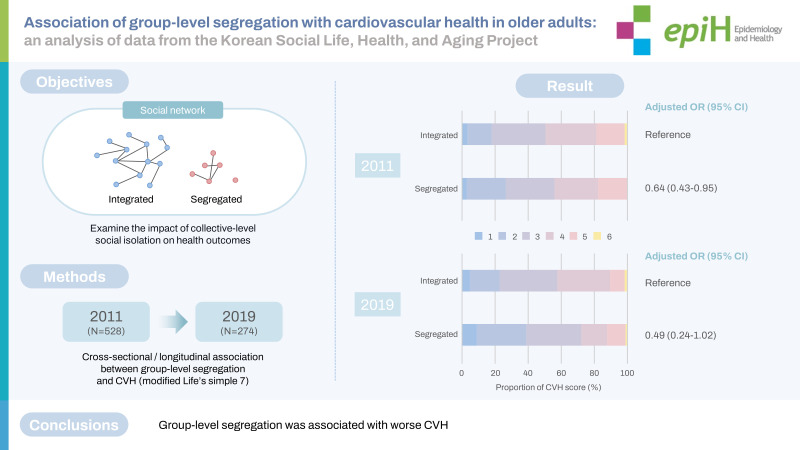


**Table 1.** Characteristics of the participants stratified by group-level segregation status at baseline

Characteristics		Total (n=528)	Integrated (n=420)	Segregated (n=108)	p-value
Age (yr)		71.7±7.4	72.0±7.0	70.3±8.4	0.025
Sex					0.360
	Male	211 (40.0)	172 (41.0)	39 (36.1)	
	Female	317 (60.0)	248 (59.0)	69 (63.9)	
Educational attainment					0.011
	Elementary school or less	168 (31.8)	144 (34.3)	24 (22.2)	
	Middle school	223 (42.2)	174 (41.4)	49 (45.4)	
	High school	71 (13.4)	58 (13.8)	13 (12.0)	
	College or higher	66 (12.5)	44 (10.5)	22 (20.4)	
Social network size		3.2±1.3	3.3±1.3	2.8±1.2	<0.001
Marital status					0.044
	Living with a spouse	392 (74.2)	320 (76.2)	72 (66.7)	
	Living without a spouse^1^	136 (25.8)	100 (23.8)	36 (33.3)	
MMSE score		24.0±4.6	24.2±4.1	23.3±6.1	0.068
Household income (US$/yr)					0.952
	<10,000	288/431 (66.8)	239/358 (66.8)	49/73 (67.1)	
	≥10,000	143/431 (33.2)	119/358 (33.2)	24/73 (32.9)	
CVH score					0.369
	6	9 (1.7)	9 (2.1)	0 (0.0)	
	5	79 (15.0)	66 (15.7)	13 (12.0)	
	4	152 (28.8)	123 (29.3)	29 (26.9)	
	3	169 (32.0)	130 (31.0)	39 (36.1)	
	2	96 (18.2)	76 (18.1)	20 (18.5)	
	1	23 (4.4)	16 (3.8)	7 (6.5)	

Values are presented as mean±standard deviation for continuous variables and number (%) for categorical variables. CVH, cardiovascular health; MMSE, Mini-Mental State Examination for Dementia Screening.

1 Including participants who were widowed (n=133) or separated (n=1), or who never married (n=2).

**Table 2.** Association of group-level segregation with cardiovascular health scores at baseline^1^

Factors		Model 1	p-value	Model 2	p-value	Model 3	p-value
Group-level segregation		0.65 (0.44, 0.95)	0.027	0.61 (0.41, 0.90)	0.013	0.64 (0.43, 0.95)	0.026
Age, per year		0.96 (0.94, 0.98)	0.001	0.95 (0.93, 0.98)	<0.001	0.96 (0.94, 0.99)	0.003
Female sex		0.88 (0.62, 1.25)	0.481	0.79 (0.54, 1.14)	0.211	0.80 (0.55, 1.16)	0.233
Educational attainment							
	Elementary school or less	1.00 (reference)		1.00 (reference)		1.00 (reference)	
	Middle school	1.16 (0.78, 1.70)	0.464	1.22 (0.82, 1.80)	0.324	1.13 (0.76, 1.68)	0.551
	High school	0.95 (0.53, 1.68)	0.858	1.00 (0.56, 1.77)	0.992	0.89 (0.50, 1.61)	0.712
	College or higher	1.11 (0.61, 2.01)	0.730	1.20 (0.66, 2.18)	0.549	1.05 (0.57, 1.95)	0.866
Social network size		-		0.96 (0.84, 1.10)	0.537	0.95 (0.83, 1.09)	0.474
Living without a spouse		-		1.41 (0.93, 2.14)	0.103	1.43 (0.94, 2.17)	0.092
MMSE score		-			-	1.04 (1.00, 1.08)	0.056

Values are presented as odds ratio (95% confidence interval). MMSE, Mini-Mental State Examination for Dementia.

1 Model 1 was adjusted for age, sex, and educational attainment; Model 2 was further adjusted for social network size and marital status; Model 3 was further adjusted for the MMSE score.

**Table 3.** Temporal changes in group-level segregation status, CVH score, and ideal CVH metrics over the 8-year follow-up (n=274)

Variables		Baseline (2011)	Follow-up (2019)	p-value
Group-level segregation		34 (12.4)	90 (32.8)	<0.001
CVH score				0.033
	6	4 (1.5)	4 (1.5)	
	5	47 (17.2)	26 (9.5)	
	4	83 (30.3)	73 (26.6)	
	3	88 (32.1)	94 (34.3)	
	2	43 (15.7)	60 (21.9)	
	1	9 (3.3)	17 (6.2)	
Ideal CVH metric				
	Smoking	248 (90.5)	256 (93.4)	0.209
	Body mass index	83 (30.3)	86 (31.4)	0.781
	Blood pressure	48 (17.5)	33 (12.0)	0.071
	Fasting glucose	209 (76.3)	182 (66.4)	0.011
	Total cholesterol	157 (57.3)	149 (54.4)	0.491
	Physical function	205 (74.8)	159 (58.0)	<0.001

Values are presented as number (%). CVH, cardiovascular health.

**Table 4.** Association of group-level segregation at baseline^1^ with CVH scores at the 8-year follow-up^2^

Factors		Model 1	p-value	Model 2	p-value	Model 3	p-value
Group-level segregation		0.56 (0.27, 1.14)	0.107	0.51 (0.25, 1.06)	0.073	0.49 (0.24, 1.02)	0.058
Age, per year		0.96 (0.92, 1.00)	0.070	0.96 (0.91, 1.00)	0.052	0.95 (0.91, 1.00)	0.036
Female sex		0.77 (0.46, 1.29)	0.318	0.72 (0.42,1.23)	0.233	0.70 (0.41, 1.20)	0.201
Educational attainment							
	Elementary school or less	1.00 (reference)	-	1.00 (reference)	-	1.00 (reference)	-
	Middle school	1.04 (0.59, 1.82)	0.897	1.07 (0.61, 1.89)	0.814	1.16 (0.64, 2.07)	0.626
	High school	0.88 (0.38, 2.02)	0.758	0.88 (0.38, 2.03)	0.761	0.96 (0.41, 2.25)	0.926
	College or higher	0.90 (0.39, 2.09)	0.807	0.96 (0.41, 2.26)	0.923	1.07 (0.45, 2.55)	0.885
Social network size		-	-	0.91 (0.76, 1.09)	0.320	0.92 (0.76, 1.10)	0.367
Living without a spouse		-	-	1.08 (0.57, 2.05)	0.811	1.06 (0.56, 2.02)	0.850
MMSE score		-	-	-	-	0.96 (0.90, 1.02)	0.220

Values are presented as odds ratio (95% confidence interval). CVH, cardiovascular health; MMSE, Mini-Mental State Examination for Dementia Screening.

1 All covariables were collected or measured at baseline.

2 Model 1 was adjusted for baseline CVH score, age, sex, and educational attainment; Model 2 was further adjusted for social network size and marital status; Model 3 was further adjusted for the MMSE score.

## SUPPLEMENTARY MATERIALS

Supplementary Material 1. Characteristics of the participants in the longitudinal analysis stratified by group-level segregation status at baseline

Supplementary Material 2. Association of group-level segregation with ideal status of each cardiovascular health metric at baseline and at 8-year follow-up

Supplementary Material 3. Characteristics of the participants who completed and were lost to follow-up
