# Supplementary material for: Association of group-level segregation with cardiovascular health in older adults: an analysis of data from the Korean Social Life, Health, and Aging Project
Source: Epidemiol Health. 2023 Apr 4;45:e2023041. doi: 10.4178/epih.e2023041 (PMC10396819; doi:10.4178/epih.e2023041)
Supplement: Supplementary Material 1. — Characteristics of the participants in the longitudinal analysis stratified by group-level segregation status at baseline [file epih-45-e2023041-Supplementary-1.docx]

**Supplemental Material 1.** Characteristics of the participants in the longitudinal analysis stratified by group-level segregation status at baseline

| Variable | Total  N=274 |  | Integrated N=240 | Segregated N=34 | p-value |
| --- | --- | --- | --- | --- | --- |
| Age, years | 69.7 ± 5.8 |  | 70.2 ± 5.6 | 66.6 ± 5.9 | <0.001 |
| Sex |  |  |  |  | 0.714 |
| Male | 105 (38.3) |  | 91 (37.9) | 14 (41.2) |  |
| Female | 169 (61.7) |  | 149 (62.1) | 20 (58.8) |  |
| Educational attainment |  |  |  |  | <0.001 |
| Elementary school or less | 85 (31.0) |  | 80 (33.3) | 5 (14.7) |  |
| Middle school | 117 (42.7) |  | 104 (43.3) | 13 (38.2) |  |
| High school | 39 (14.2) |  | 35 (14.6) | 4 (11.8) |  |
| College or higher | 33 (12.0) |  | 21 (8.8) | 12 (35.3) |  |
| Social network size | 3.3 ± 1.3 |  | 3.4 ± 1.3 | 3.0 ± 0.9 | 0.070 |
| Marital status |  |  |  |  | 0.067 |
| Living with a spouse | 218 (79.6) |  | 190 (79.2) | 28 (82.4) |  |
| Living without a spouse^*^ | 56 (20.4) |  | 50 (20.8) | 6 (17.6) |  |
| MMSE score | 25.0 ± 3.6 |  | 25.0 ± 3.3 | 25.0 ± 5.4 | 0.099 |
| Household income |  |  |  |  | 0.162 |
| < $10,000 per year | 147/231 (63.6) |  | 136/209 (65.1) | 11/22 (50.0) |  |
| ≥ $10,000 per year | 84/231 (36.4) |  | 73/209 (34.9) | 11/22 (50.0) |  |
| CVH score |  |  |  |  | 0.794 |
| 6 | 4 (1.5) |  | 4 (1.7) | 0 (0.0) |  |
| 5 | 47 (17.2) |  | 41 (17.1) | 6 (17.6) |  |
| 4 | 83 (30.3) |  | 74 (30.8) | 9 (26.5) |  |
| 3 | 88 (32.1) |  | 78 (32.5) | 10 (29.4) |  |
| 2 | 43 (15.7) |  | 35 (14.6) | 8 (23.5) |  |
| 1 | 9 (3.3) |  | 8 (3.3) | 1 (2.9) |  |

Values as mean ± standard deviation for continuous variables and number (%) for categorical variables.

^*^Including participants who were widowed (N=55) or separated (N= 1).
