# Supplementary material for: Association of group-level segregation with cardiovascular health in older adults: an analysis of data from the Korean Social Life, Health, and Aging Project
Source: Epidemiol Health. 2023 Apr 4;45:e2023041. doi: 10.4178/epih.e2023041 (PMC10396819; doi:10.4178/epih.e2023041)
Supplement: Supplementary Material 2. — Association of group-level segregation with ideal status of each cardiovascular health metric at baseline and at 8-year follow-up [file epih-45-e2023041-Supplementary-2.docx]

**Supplemental Material 2.** Association of group-level segregation with ideal status of each cardiovascular health metric at baseline and at 8-year follow-up

| Metric | Baseline (2011) | | |  | Follow-up (2019) | | |
| --- | --- | --- | --- | --- | --- | --- | --- |
|  | OR | (95% CI) | p-value |  | OR | (95% CI) | p-value |
| Smoking | 1.06 | (0.47-2.41) | 0.887 |  | 1.37 | (0.23-7.96) | 0.729 |
| Body mass index | 0.76 | (0.47-1.24) | 0.269 |  | 0.71 | (0.21-2.38) | 0.583 |
| Blood pressure | 0.95 | (0.52-1.74) | 0.870 |  | 0.87 | (0.25-3.05) | 0.827 |
| Fasting glucose | 0.73 | (0.45-1.19) | 0.204 |  | 0.35 | (0.14-0.89) | 0.027 |
| Total cholesterol | 1.23 | (0.78-1.94) | 0.374 |  | 0.54 | (0.23-1.24) | 0.147 |
| Physical function | 0.55 | (0.32-0.94) | 0.030 |  | 0.82 | (0.28-2.42) | 0.725 |

The models were adjusted for age, sex, educational attainment, social network size, marital status, and Mini-Mental State Examination for Dementia score. In the analyses for follow-up metrics, the models were additionally adjusted for the baseline ideal status of each metric.
