# Supplementary material for: Association of group-level segregation with cardiovascular health in older adults: an analysis of data from the Korean Social Life, Health, and Aging Project
Source: Epidemiol Health. 2023 Apr 4;45:e2023041. doi: 10.4178/epih.e2023041 (PMC10396819; doi:10.4178/epih.e2023041)
Supplement: Supplementary Material 3. — Characteristics of the participants who completed and were lost to follow-up [file epih-45-e2023041-Supplementary-3.docx]

**Supplemental Material 3.** Characteristics of the participants who completed and were lost to follow-up

| Variable | Completed follow-up  N=274 | Were lost to follow-up  N=254 | p-value |
| --- | --- | --- | --- |
| Group-level segregation | 34 (12.4) | 74 (29.1) | <0.001 |
| Age | 69.7 ± 5.8 | 73.8 ± 8.3 | <0.001 |
| Female | 169 (61.7) | 148 (58.3) | 0.084 |
| CVH score |  |  | 0.495 |
| 6 | 4 (1.5) | 5 (2.0) |  |
| 5 | 47 (17.2) | 32 (12.6) |  |
| 4 | 83 (30.3) | 69 (27.2) |  |
| 3 | 88 (32.1) | 81 (31.9) |  |
| 2 | 43 (15.7) | 53 (20.9) |  |
| 1 | 9 (3.3) | 14 (5.5) |  |

Values as mean ± standard deviation for continuous variables and number (%) for categorical variables.

CVH, cardiovascular health.
